# Recent and rapid ecogeographical rule reversals in Northern Treeshrews

**DOI:** 10.1038/s41598-022-23774-w

**Published:** 2022-11-29

**Authors:** Maya M. Juman, Virginie Millien, Link E. Olson, Eric J. Sargis

**Affiliations:** 1 Department of Ecology and Evolutionary Biology, Yale University, New Haven, CT USA; 2 Department of Mammalogy, University of Alaska Museum, University of Alaska Fairbanks, Fairbanks, AK USA; 3 Department of Veterinary Medicine, University of Cambridge, Cambridge, UK; 4 Redpath Museum, McGill University, Montreal, QC Canada; 5 Department of Anthropology, Yale University, New Haven, CT USA; 6 Divisions of Vertebrate Zoology and Vertebrate Paleontology, Yale Peabody Museum of Natural History, New Haven, CT USA; 7 Yale Institute for Biospheric Studies, New Haven, CT USA

**Keywords:** Biogeography, Climate-change ecology, Evolution, Zoology

## Abstract

Two of the most-studied ecogeographical rules describe patterns of body size variation within species. Bergmann’s rule predicts that individuals have larger body sizes in colder climates (typically at higher latitudes), and the island rule predicts that island populations of small-bodied species average larger in size than their mainland counterparts (insular gigantism). These rules are rarely tested in conjunction or assessed across space and time simultaneously. We investigated these patterns in the Northern Treeshrew (*Tupaia belangeri*) using museum specimens collected across a wide spatial and temporal range. Contrary to Bergmann’s rule, size increases with temperature in *T. belangeri*, a signal that is highly consistent across space and time. We also show that these rules are intertwined: Bergmann’s rule is reversed on the mainland but holds on islands, and therefore the island rule is upheld at higher, but not lower, latitudes. Moreover, we demonstrate a rapid reversal of both rules over time. The mechanism behind these inversions remains unclear, though temperature and precipitation are significant predictors of body size. Ecogeographical rules rely on the assumption of a constant relationship between size and the factors driving its variation. Our results highlight the need to question this assumption and reevaluate these rules in the context of accelerating and uneven climate change.

Few ecogeographical patterns are as important, conspicuous, and well studied as intraspecific variation in animal body size^1^. Body size is associated with a number of critical physiological, ecological, and behavioral traits^2^ and is known to vary based on environmental and geographical factors. Consequently, there are several well-known “rules” that are thought to govern body size variation. Perhaps the most widely studied is Bergmann’s rule, which describes a pattern of larger body size in individuals at higher latitudes (colder climates) relative to those at lower latitudes (warmer climates), owing to the advantages of minimizing heat loss and cooling costs, respectively^3–5^. Another well-studied rule is the island rule, which refers to the tendency of large-bodied species (> 5 kg) to evolve smaller size on islands (insular dwarfism), while small-bodied species are larger on islands than on the mainland (insular gigantism)^6^. Both rules are generally well supported across a variety of taxonomic groups^1,7,8^, although exceptions apply^9,10^. However, the possible interaction between these rules is unclear, as they are rarely tested in conjunction^11–14^.

Because Bergmann’s rule hinges on a negative relationship between temperature and body size, it provides a theoretical framework for predicting species’ responses to ongoing climate change^1^. In mammals, morphological changes are the first response to global warming, and these changes are detectable within 100 years^15^. Many have hypothesized that species will decrease in size as temperatures rise^16,17^, and some studies have established this pattern in both contemporary and historical records^1^. However, it is unclear whether this is a universal trend across taxa and whether it represents an adaptive response, phenotypic plasticity, or a combination thereof^18,19^. The exact mechanism behind Bergmann’s rule is also unresolved; although temperature remains the most obvious explanation, other (related) factors may also explain patterns in body size, such as precipitation and primary productivity^20–22^. Moreover, no studies have investigated whether the trends predicted by ecogeographical rules remain consistent over time in their direction and strength, especially as climate change differentially impacts various regions and ecosystems.

Here, we simultaneously examine Bergmann’s rule and the island rule in a small Southeast Asian mammal with a wide latitudinal range across the tropics and subtropics (Fig. 1), the Northern Treeshrew, *Tupaia belangeri* (Wagner, 1841)^23^. This species averages about 180 g, is known to feed on invertebrates and fruit, and inhabits a variety of habitats, including degraded forests^24^. With a broad distribution on islands and the mainland, poor overwater dispersal ability, and a relatively short generation time^24,25^, the Northern Treeshrew provides an excellent system for testing ecogeographical rules. In experimental studies of captive individuals, seasonal weight fluctuations in this species appeared to be consistent with Bergmann’s rule, with body weight increasing under winter-like conditions^26,27^. However, it is not known if its body size varies similarly along temperature gradients in the wild. Here, we tested this hypothesis, as well as the validity of both Bergmann’s rule and the island rule, in *T. belangeri*. We collected a large data set from museum specimens acquired over 130 years, across the entire latitudinal and altitudinal distribution, and from both mainland and island populations. Our study examined (1) whether the Northern Treeshrew conforms to ecogeographical rules of body size variation across both spatial and temporal scales, and (2) if the pattern in size variation observed today is consistent in magnitude and direction over time. We further explored environmental factors that have changed over time in the region and how they relate to size variation in *T. belangeri*.

**Figure 1:**
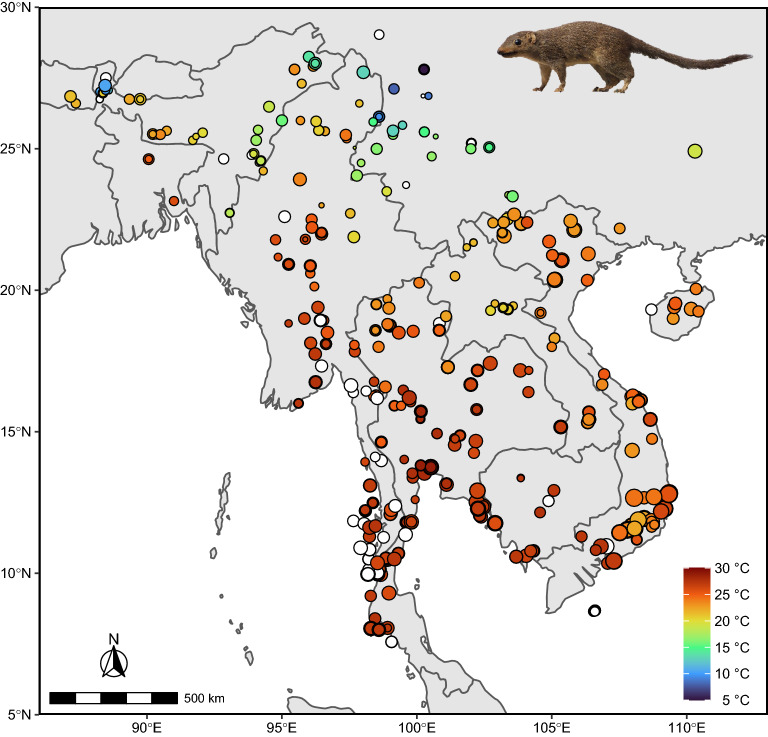
Locality map of specimens included in our study. Point size corresponds to relative size of individual based on Principal Component 1 (Supplementary Table S1). Point color reflects the average annual temperature at each locality in the year when the specimen was collected. White circles indicate localities for which we do not have historical climate data for the collection year. Map was generated using the packages *ggplot2*^28^, *rnaturalearth*^29^, and *ggspatial*^30^ in R (version 3.5.1)^31^. Image in the upper right corner depicts a Northern Treeshrew (photo by Daderot, CC0 1.0, Wikimedia Commons).

## Results

Before examining ecogeographical rules, we assessed intraspecific morphometric variation in *T. belangeri*, a species with a complex taxonomic history. Our investigation revealed complete overlap among subspecies (Supplementary Fig. S1; Supplementary Information), including *T. b. chinensis*, which was previously recognized by Helgen^32^. Given that there is no evidence for skull shape or size variation among these populations, we treated our entire sample as a single taxon.

The first principal component (PC1) in our PCA of the complete imputed data set explained over 75% of the variation with high positive loadings across all 18 cranial and mandibular variables (Supplementary Table S1), so higher PC1 values indicate larger skull size. PC1 was also positively correlated with total body length, body weight, and skull length (Pearson correlation coefficient *r* = 0.47, 0.58, 0.98, respectively; all *p* < 0.0001). Therefore, we use PC1 as a proxy for body size in all subsequent analyses.

We found significant sexual size dimorphism in our sample, with males having higher PC1 scores than females (*t* = − 7.6, *p* < 0.0001). Sex was therefore included in all analyses to control for sexual dimorphism.

### Bergmann’s rule and the island rule are intertwined

**Figure 2:**
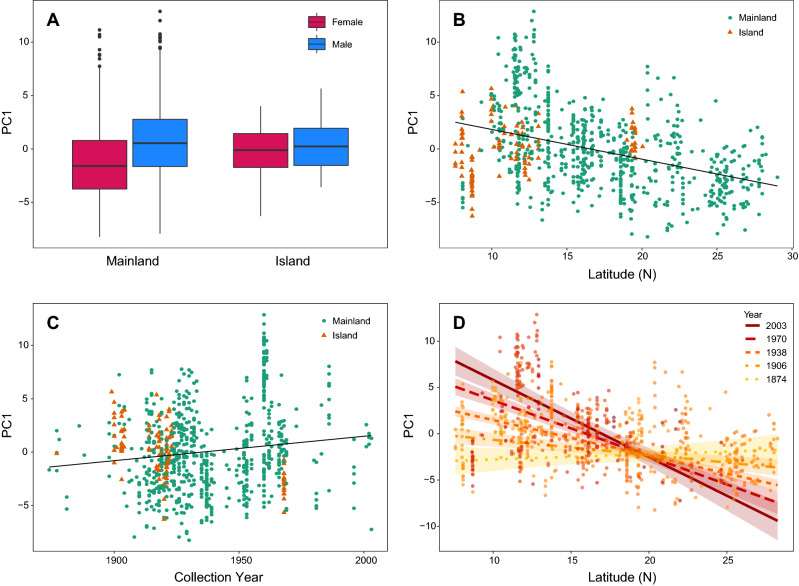
Spatial and temporal patterns in *T. belangeri* body size (Supplementary Table S2). (**A**) Box plots of females and males from the mainland and islands show mainland populations have a much greater degree of sexual size dimorphism than island populations, as suggested by a significant interaction between Sex and Source (Mainland/Island) in our model (*p* < 0.05). (**B**) Linear relation between PC1 and Latitude shows that there is a reversal of Bergmann’s rule (decreasing body size with latitude) on the mainland (*p* < 0.0001)*,* though the smaller island sample shows a slight increase at higher latitudes (*p* < 0.0001). Consequently, Foster’s island rule may be upheld at higher latitudes (insular gigantism in these small mammals) more than at lower latitudes. (**C**) Linear relation between PC1 and Collection Year shows that there is a significant increase in size (PC1) throughout time (*p* < 0.0001). (**D**) A plot depicting the positive interaction (*p* < 0.0001) between Latitude and Collection Year in a linear model predicting body size (PC1). Regression lines, displayed at five evenly spaced intervals across the temporal range of the sample and plotted with 95% confidence intervals, indicate a reversal of Bergmann’s rule over time.

We ran backward stepwise selection on a linear model with PC1 as a response variable and Sex, Latitude, Source (Mainland/Island), and Collection Year as predictors, including all possible interaction terms. Our final model (adjusted R^2^ = 0.350) revealed that sexual size dimorphism varies between mainland and island populations (Supplementary Table S2). On the mainland, males were significantly larger than females, as expected (*t* = 9.2, *p* < 0.0001), but this difference was nearly erased on islands (*p* < 0.05; Fig. 2A); there was no interaction term between Sex and Latitude included in the final model. Overall, size decreased with increasing latitude (*t* = − 16.0, *p* < 0.0001; Fig. 2B). However, a significant interaction between Latitude and Source (*p* < 0.0001) indicated that on islands, body size increases with latitude. As a result, island individuals average larger than mainland individuals at higher latitudes and smaller than mainland individuals at lower latitudes (Fig. 2B). There is no significant island effect overall (*p* = 0.58). The directions and significance levels of these predictors and their interaction terms were consistent across models run on the overall dataset, male sample, and female sample, so only the overall model is reported here.

### Rule reversals over time

Our linear model also revealed several temporal trends in body size (Supplementary Table S2). Overall, PC1 increased with later collection years (*t* = 4.9, *p* < 0.0001; Fig. 2C). Island individuals show the opposite pattern of decreasing in size over time, but this effect was not significant (*p* = 0.12). There was also no apparent change in sexual size dimorphism across time, so this interaction was excluded from our final model. On the mainland, the relationship between latitude and body size has become more steeply negative over time, a gradual reversal of Bergmann’s rule (*t* = − 6.5, *p* < 0.0001; Fig. 2D). However, the opposite trend is present in island populations, as suggested by a significant three-way interaction between Source, Collection Year, and Latitude (*t* = 2.39, *p* < 0.05). Our island results must be interpreted with caution due to uneven sampling of island individuals across the latitudinal and temporal range of our data set, as evidenced by the wide confidence intervals for the island group relative to the mainland sample (Supplementary Fig. S2).

### Distance from mainland is the major driver of island body size

**Figure 3:**
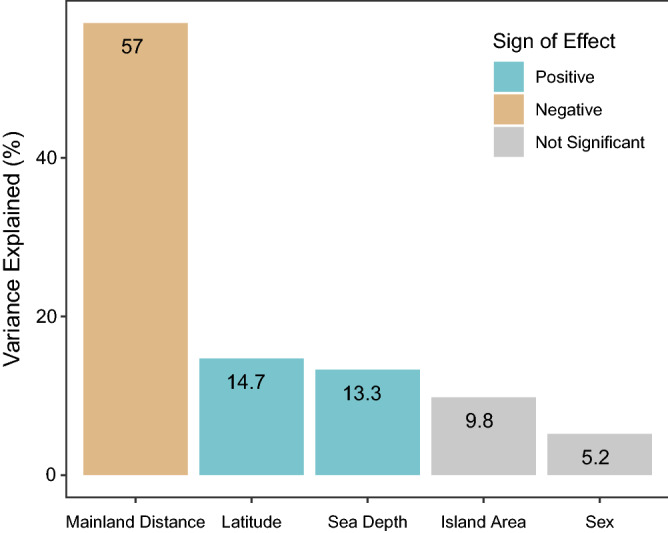
Percentage of variance explained by each variable included in a hierarchical partitioning analysis of body size on islands (Supplementary Table S3). Colors of bars correspond to the sign of the relationship between each variable and body size, as determined by a separate linear model and individual Pearson correlation tests. Variables identified as non-significant contributors to body size are indicated with gray bars.

Because Island Area was correlated with Sea Depth, Latitude, and Mainland Distance (Pearson correlation coefficient *r* = 0.40, 0.82, − 0.21 and *p* < 0.0001, 0.0001, 0.05, respectively), and Sea Depth was also correlated with Latitude and Mainland Distance (Pearson correlation coefficient *r* = 0.63, 0.62, respectively; both *p* < 0.0001), we used a hierarchical partitioning analysis to examine the effect of these factors, as well as Sex, on island body size. It revealed that Mainland Distance (independent contribution of 57.0%; Z = 13.56, *p* < 0.05) explained over half the variance in body size on islands. Latitude (independent contribution of 14.7%; Z = 2.74, *p* < 0.05) and Sea Depth (independent contribution of 13.3%; Z = 2.42, *p* < 0.05) accounted for additional variance, but neither Island Area (independent contribution of 9.8%; Z = 1.64, *p* > 0.05) nor Sex (independent contribution of 5.2%; Z = 0.52, *p* > 0.05) were significant factors (Fig. 3; Supplementary Table S3).

Correlation tests and a linear model with these variables indicated that PC1 scores for our island sample decreased with increasing Mainland Distance but increased with Latitude and Sea Depth. Therefore, *T. belangeri* individuals are larger on more northern islands (consistent with the latitudinal trend observed in Fig. 2B), but size variation on islands was mostly driven by a negative relationship between distance from the mainland and body size. Sex does not appear to play a significant role in determining island body size (consistent with Fig. 2A).

### Environmental variables underlying body size variation across space and time

**Figure 4:**
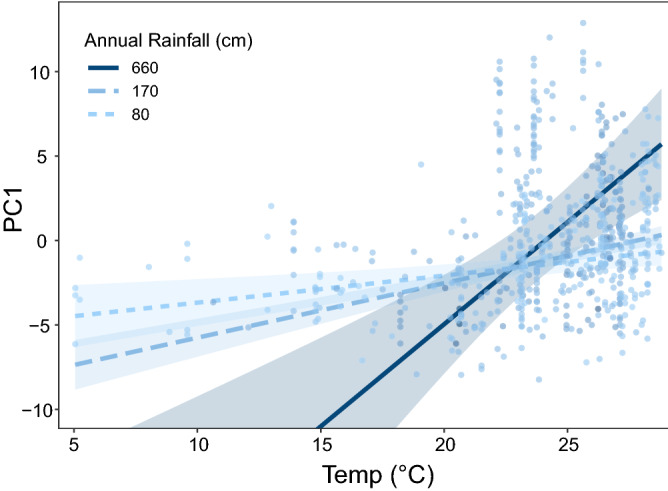
A plot depicting the positive interaction between average annual temperature and annual total rainfall in a linear model predicting body size (PC1) (Supplementary Table S4). Regression lines are displayed at the approximate minimum, median, and maximum rainfall values and plotted with 95% confidence intervals.

We ran a spatial lag regression on PC1 including environmental variables for which historical data were available (Temperature, Precipitation, Elevation, Primary Forest Cover, Total Forest Cover, and Urban Proximity), as well as Sex. The resulting model had an AIC of 2997.7 (relative to an original AIC of 3490.5 in a linear regression) and residual spatial autocorrelation was non-significant. Sex was statistically significant (*z* = 11.8, *p* < 0.0001), with males being larger than females. Additionally, *T. belangeri* increases in size with temperature (*z* = 3.0, *p* < 0.01), and a positive interaction between Temperature and Precipitation (*z* = 2.1, *p* < 0.05) indicates that in wetter climates, the positive relationship between temperature and body size is steeper (Fig. 4). All other predictors were not statistically significant (Supplementary Table S4).

## Discussion

Our study revealed a number of unexpected body size trends in Northern Treeshrews. As suggested in an earlier study, *T. belangeri* appears to be sexually dimorphic in size^33^, but only on the mainland (Figs. 2A and 3). Bergmann’s rule is reversed in this species, with individuals at higher latitudes being smaller in size than those closer to the equator (Figs. 1 and 2B). There is no evidence for an overall island effect on body size (Supplementary Table S2). Furthermore, contrary to other studies in mammals^7,14,34^, island area does not play a significant role in determining island body size (Fig. 3). Instead, distance to the nearest mainland explains the most variance in body size, but in the opposite direction than expected^8^, with smaller individuals on more distant islands (Fig. 3). The islands in our study included continental islands connected to the mainland until 400 ka^35^ (e.g., Côn Sơn) as well as larger islands separate from the Sunda shelf (e.g., Hainan). Sea depth, often used as a proxy for the length of isolation from the mainland (Supplementary Table S6), was positively associated with body size and explained a significant amount of variance (Fig. 3).

Although previous studies have found that Bergmann’s rule or the island rule are inverted or inapplicable in other treeshrew species^14,36,37^, ours is the first to demonstrate an interaction between these two rules in this group of mammals, and one of the very few to show this in any mammalian species^11,12,38^. Mainland individuals of *T. belangeri* display a clear reversal of Bergmann’s rule, whereas island individuals increase in size with latitude as expected (Figs. 2B, 3; Supplementary Table S2). Thus, the island rule may be more applicable at higher latitudes, where island individuals average larger than those on the mainland, but it is reversed at lower latitudes (Fig. 2B). This interplay between rules highlights the fact that body size is linked to a complex array of dynamic and potentially interdependent ecological and geographical factors that cannot be encapsulated in a single variable. Our study emphasizes the necessity of integrative analyses that test these rules in conjunction.

We discovered a significant positive temporal trend in body size, with *T. belangeri* generally getting larger over time on the mainland (Fig. 2C). We found that temperature was the most important predictor of body size after sex, but in the opposite direction as hypothesized: Northern Treeshrews are larger at localities with higher temperatures (Fig. 1, Supplementary Table S4). This pattern was highly consistent across space (larger at lower latitudes; Figs. 1 and 2B; Supplementary Table S2) and time (larger in more recent, warmer years; Fig. 2C; Supplementary Table S2). Another factor that might contribute to body size is precipitation; in areas with heavier rainfall, the relationship between temperature and body size is even more steeply positive (Fig. 4; Supplementary Table S4). This observation is congruent with earlier studies that have suggested a temperature-rainfall modification to Bergmann’s rule as a better predictor of body size than temperature alone^20–22,39^. Despite extensive land-use change in the region (Supplementary Fig. S3), neither forest cover nor urbanization were significant predictors of body size (Supplementary Table S4).

The mean annual temperature across all localities in our study was 24.14 °C from 1901 to 1905 (Supplementary Fig. S3) but has risen to 24.99 °C (a 1.53 °F increase) in 2020, the most recent year on record. Decreases in body size are not a universal response to global warming, and size increases have been noted in other mammalian species^40,41^. However, those species are endemic to higher latitudes, where larger individuals may have a selective advantage in handling extreme seasonal fluctuations. The Northern Treeshrew is distributed across the tropics and subtropics, and thus the mechanism here remains unclear. Given that body mass in individuals of this species has been shown to fluctuate annually in a pattern consistent with Bergmann’s rule^26,27^, our study suggests that temperature is likely not the only influential factor in the wild.

Most importantly, we demonstrated that both rules have inverted over a surprisingly shallow temporal scale. Bergmann’s rule shows a clear trend of reversing and becoming more steeply reversed over time (Fig. 2D). In the late nineteenth century there was a positive relationship between latitude and body size, as Bergmann himself suggested in 1847^3^. By the twentieth century the slope had reversed, and in more recent years has become increasingly negative. On islands, the latitudinal slope has moved in the opposite direction (Supplementary Fig. S2), and this has contributed to a reversal of the island rule over time. Although island individuals collected in the late 19th and early twentieth centuries were generally larger than mainland individuals as expected, they averaged smaller body size during later collection years (Supplementary Table S2; Fig. 2C; Supplementary Fig. S2). However, this result is not statistically significant and is based on a relatively small island sample (*n* = 111) and sporadic island collection trips. More recent data are required from island surveys across a wider latitudinal range to test this trend. Additionally, we were not able to analyze environmental factors that could be contributing to the reversed body size trends on islands due to the lack of historical data from many of the smaller, distant islands in our sample.

The reversal of the rules may be due to the fact that climate change and other anthropogenic impacts manifest in different ways across regions; for example, higher latitudes experience greater magnitudes of warming, whereas tropical biodiversity is most heavily impacted due to narrower ranges of thermal tolerance^42^ and greater rates of land use^43^. Islands may be buffered against extreme climate change by surrounding oceans^44^ but are at higher risk of habitat loss relative to mainland regions^45^. Thus, ecogeographical rules should be revisited in the context of ongoing global change, with temporal and ideally climatic variables included in studies of different taxa. Much attention has been paid to using rules to predict species’ responses to rising temperatures^1,13^, but to our knowledge no studies have explored the possibility that climate change may be rewriting the rules themselves.

Generally, omitting time from analyses of body size may obscure patterns caused by temperature, rainfall, and other rapidly shifting environmental and anthropogenic factors. For example, an analysis of Bergmann’s rule that does not account for non-random sampling across time and space (e.g., if all northern samples were collected a century before the southern samples) could reveal a body size gradient that amounts to a statistical artifact. Similarly, uneven temporal sampling may mask more complex patterns occurring at and around an inflection point in time, especially when treating time as a categorical rather than continuous variable. This study is rare among analyses of body size and climate change as it includes a large sample size with a continuous temporal distribution, thereby avoiding these potential pitfalls^46^. Indeed, of the 27 such studies recently reviewed by Theriot et al.^47^, only one involved a larger sample of conspecifics (but across only 30 years of sampling), and none spanned as extensive a temporal range for a single species.

Finally, like many other studies of ecogeographic variation, ours relies entirely on an invaluable data set of specimens spanning a broad temporal and spatial range. Museum collection efforts have stalled in recent decades, creating major gaps in our record of species amidst unprecedented global change^48^. Our work highlights the crucial ongoing role of museums in the documentation, prediction, and mitigation of anthropogenic impacts on biodiversity.

## Methods

### Data collection

EJS recorded 22 craniomandibular measurements (Supplementary Table S5)^14,36,49–54^ from 839 adult (those with fully erupted permanent dentition^55^) museum specimens of *Tupaia belangeri* (Supplementary Appendix) using Mitutoyo digital calipers that read to 0.01 mm. Because of occasional broken or damaged skulls, we were unable to record all measurements for all individuals. Examined specimens are housed in the following museums: American Museum of Natural History (AMNH), New York, NY; Carnegie Museum of Natural History (CMNH), Pittsburgh, PA; Field Museum of Natural History (FMNH), Chicago, IL; KU Biodiversity Institute & Natural History Museum (KU), Lawrence, KS; Natural History Museum of Los Angeles County (LACM), Los Angeles, CA; Museum of Comparative Zoology at Harvard University (MCZ), Cambridge, MA; Muséum national d’Histoire naturelle (MNHN), Paris, France; Museum of Vertebrate Zoology (MVZ), Berkeley, CA; The Natural History Museum (NHMUK), London, UK; Royal Ontario Museum (ROM), Toronto, ON; Senckenberg Naturmuseum (SMF), Frankfurt, Germany; University of Michigan Museum of Zoology (UMMZ), Ann Arbor, MI; United States National Museum of Natural History (USNM), Washington, DC; Yale Peabody Museum of Natural History (YPM), New Haven, CT; Museum für Naturkunde (ZMB), Berlin, Germany; and Zoologisk Museum University of Copenhagen (ZMUC), Copenhagen, Denmark.

Our sample included both currently recognized subspecies of *T. belangeri*: *T. b. belangeri* (*n* = 744) and *T. b. chinensis* (*n* = 95), as well as 20 additional previously recognized taxa now synonymized with *T. b. belangeri*^32^ (see Supplementary Information). The specimens originated from 293 distinct localities that ranged from 7.58° N to 29.03° N in latitude (Fig. 1), which we georeferenced using Google Earth (version 9.156.0.0). Two specimens could not be georeferenced due to missing locality details (ZMB 87167 and NHMUK 62.7.16.12). Most localities were on the mainland (*n* = 726)*,* and the remainder (*n* = 111) were from 21 islands (Supplementary Table S6). All but 32 specimens were associated with a date of collection, ranging from 1874 to 2003. EJS also recorded total length and body weight from specimen tags when available.

### Environmental data

For localities that did not include altitude, we obtained the elevation (m) using the *rgbif* R package^56^ at a resolution of 90 × 90 m (http://www.geonames.org/). We used the collection year to associate the following raster-based historical environmental data with each of our localities, at a gridded 0.5° × 0.5° resolution: average annual temperature (Celsius), annual total rainfall (cm/year), and percentage of total forest cover, primary forest cover, and urban land cover. Climate variables for specimens collected after 1901 were obtained from the Climatic Research Unit Time-Series 4.05^57^, and estimated land cover was obtained from a model reconstruction based on the HYDE 3.1 database^58^. Absolute urban land coverage is low across all years, and the urban cover variable was used as a proxy for general proximity to urban spaces. Because *T. belangeri* is known to occur in primary, secondary, evergreen, and deciduous forests^24^, we aggregated these forest types to include the overall percentage of forest cover in our analyses, in addition to primary forest cover alone. These environmental variables could not be associated with all island localities due to missing data from distant islands. For our island subset (*n* = 111), we obtained island area (km^2^) and distance to mainland (km) (Google Earth version 9.156.0.0; http://islands.unep.ch/isldir.htm) as well as maximum sea depth (m) (GeoMapApp 3.6.14^59^) (Supplementary Table S6). All continuous variables were ln-transformed and scaled (centered at zero) prior to inclusion in analyses.

### Taxonomic boundaries

We first conducted an analysis of taxonomic boundaries within *T. belangeri* using the original data set. We performed a principal component analysis (PCA) of eight log-transformed skull variables on 21 taxa (both previously and currently recognized subspecies). Additional details are provided in the Supplementary Methods. Based on the results of our taxonomic study, all taxa were pooled in subsequent analyses.

### Imputing missing data

Because some skulls were broken or damaged, our data set included a significant amount of missing data (18.5%), and only 225 specimens had all 22 measurements. We first removed the four variables with the most missing data (CIL, EPL, EB, and LB; see Supplementary Table S5 for abbreviation definitions), leaving us with 15.0% missing data in the remaining 18 variables and 378 individuals with the full complement of measurements. The missing cases were then imputed using the *mice* package^60^ with predictive mean matching, averaging values from 100 imputation iterations^14,61^. We also excluded one specimen (NHMUK 6.11.6.3) with many missing measurements that could not be consistently imputed and the two specimens without coordinates (ZMB 87167 and NHMUK 62.7.16.12). All original and imputed measurements are provided in Supplementary Data S1.

Next, we performed a PCA on the 18 imputed, log-transformed craniomandibular variables (Supplementary Table S1). We retained the first component (PC1) to use as a proxy for body size in subsequent analyses, all of which were conducted in R^31^.

### Statistical analyses

We first compared males and females with Welch two-sample t-tests on PC1 in the overall data set and included sex in all subsequent analyses to account for sexual size dimorphism. All linear models described below were fitted using ordinary least squares (OLS) and evaluated for fit using diagnostic plots (residual plots and q-q plots). Multicollinearity was assessed by checking for variance inflation factors (VIF) below a maximum threshold of 10 for significant interaction terms and 2.5 for significant individual predictors.

To test for Bergmann’s rule and the island rule simultaneously, as well as examine temporal trends, we ran a full linear model on the entire imputed sample (*n* = 836), using Latitude, Source (Mainland/Island), Sex, and Collection Year as predictors of body size (PC1). We included interaction terms between all predictors to investigate the possible interplay of these factors in explaining body size variation. We then used backward stepwise selection to obtain the best model using the Akaike information criterion (AIC). We ran this model on the overall dataset as well as separate samples for each sex, using PC1 generated from a PCA of each sample.

We further explored the effects of additional geographic factors on body size in island individuals (*n* = 111); these factors included distance to nearest mainland, maximum sea depth, and island area. Mainland Distance and Sea Depth are proxies for geographical and temporal isolation, respectively. Because these variables are significantly correlated with one another and with Latitude, we used a hierarchical partitioning analysis to determine the independent contributions of each of these factors, as well as Sex, on body size (PC1). We conducted our analysis using the *hier.part* package and obtained Z scores from 10,000 independent randomizations^62^. Because this method does not reveal the sign of the effects, we ran a separate linear model as well as individual Pearson correlation tests to identify the sign (positive or negative) of the effect of each factor identified as significant in the hierarchical partitioning analysis.

Finally, we explored the possible ecological factors associated with spatial and temporal patterns in body size variation, using a subset of individuals for which environmental data were available (*n* = 672). A Moran’s I test revealed statistically significant spatial autocorrelation in this subset. To account for this bias, we ran a spatial lag model using the *spatialreg* package^63^ with K-nearest neighbor weights (K = 5). We used this model to predict PC1 with Sex, Temperature, Precipitation, Elevation, Primary Forest Cover, Total Forest Cover, and Urban Proximity, as well as an interaction term between Temperature and Precipitation. We were unable to include Source in this analysis because of the lack of environmental data from smaller, distant islands. We also ran a spatial error model with the same combination of predictors but used the spatial lag model as it was a better fit for our data and had a lower AIC.

## Supplementary Information

Supplementary Information 1.

Supplementary Information 2.

## Data Availability

All measurements and associated museum catalog numbers used in these analyses are provided in the Supplementary Data S1. All R code is available on GitHub at https://github.com/mayajuman/belangeri.
