# Supplementary material for: Recent and rapid ecogeographical rule reversals in Northern Treeshrews
Source: Sci Rep. 2022 Nov 29;12:19689. doi: 10.1038/s41598-022-23774-w (PMC9708835; doi:10.1038/s41598-022-23774-w)
Supplement: Supplementary file 2 — Supplementary Information 2. [file 41598_2022_23774_MOESM2_ESM.pdf]

Supplementary Information

Recent and rapid ecogeographical rule reversals in Northern Treeshrews

Maya M. Juman[1,2,3]*, Virginie Millien[4], Link E. Olson[2], Eric J. Sargis[1,5,6,7]

[1]Department of Ecology and Evolutionary Biology, Yale University, New Haven, CT, USA; [2]Department of Mammalogy, University of Alaska Museum, University of Alaska Fairbanks, Fairbanks, AK, USA; [3]Department of Veterinary Medicine, University of Cambridge, Cambridge, UK; [4]Redpath Museum, McGill University, Montreal, QC, Canada; [5]Department of Anthropology, Yale University, New Haven, CT, USA; [6]Divisions of Vertebrate Zoology and Vertebrate Paleontology, Yale Peabody Museum of Natural History, New Haven, CT, USA; [7]Yale Institute for Biospheric Studies, New Haven, CT, USA

Table of Contents for Supplementary Information

Supplementary Taxonomic Background (p. 2)
Supplementary Methods (pp. 2–3)
Supplementary Results and Discussion (pp. 3–4)
Supplementary References (pp. 4–6)
Appendix of specimens examined (pp. 7–11)
Supplementary Tables S1–7 (pp. 12–16)
Supplementary Figures S1–3 (pp. 17–20)

Supplementary Taxonomic Background

The Northern Treeshrew has a complicated taxonomic history with 27 synonyms, which must be addressed before ecogeographic rules are tested within this species. Many of these subspecies were originally described based on subtle variation in pelage, a plastic feature that has proven unreliable for distinguishing treeshrew subspecies[1,2]. Helgen (2005: 105)[3] recognized only two subspecies—*T. b. belangeri* (Wagner, 1841)[4] and *T. b. chinensis* Anderson, 1879[5]—but he stated that "[a] careful revision of geographic variation within *T. belangeri* is needed." Additionally, *T. belangeri* was previously included in a species complex with *Tupaia glis*, the Common Treeshrew, leading to debate over the affiliation of a number of populations near the contact zone at the Isthmus of Kra[6]. However, these species differ in their mammae formula, which appears to be a fixed feature in treeshrews[7-9]; *T. glis* has only four mammae whereas *T. belangeri* has six[7]. For populations close to the contact zone, we use this discrete character, rather than latitudinal range, to differentiate between species. Therefore, we include in our dataset *T. "glis" cognata*, a taxon with six mammae, and exclude *T. "belangeri" kohtauensis*, *T. "belangeri" operosa*, and *T. "belangeri" ultima*, which have four mammae.

We examined infraspecific variation within *T. belangeri* using morphometric data from the skulls of 839 museum specimens, a method that we have previously used to delineate treeshrew species and subspecies[1,2,8-12]. We assessed the degree of morphometric separation among the 22 putative taxa in our dataset, as well as between the two currently recognized subspecies.

Supplementary Methods

To examine craniomandibular variation among the subspecies of *T. belangeri*, we used R with function princomp() and package *ggplot2*[13,14] to conduct and plot a principal component analysis

(PCA). Missing data prevented the inclusion of all the specimens and variables. We followed earlier studies of *T. hypochrysa*, *T. palawanensis*, *T. chrysogaster*, *T. ferruginea*, *T. glis*, *T. tana*, *T. minor*, and *Ptilocercus lowii* in that we did not impute missing data for this taxonomic analysis[1,2,7-12]. Therefore, eight log-transformed variables were selected based on their completeness in the sample in order to optimize the number of individuals included: UTL, PBPL, LTPL, MTL, LIB, MH, MCH, and MCW (abbreviations defined in Supplementary Table S5). Our sample for this PCA included 21 names ($n = 569$): *T. b. annamensis* Robinson and Kloss, 1922[15] ($n = 43$); *T. b. assamensis* Wroughton, 1921[16] ($n = 14$); *T. b. belangeri* (Wagner, 1841)[4] ($n = 18$); *T. b. brunetta* Thomas, 1923[17] ($n = 10$); *T. b. cambodiana* Kloss, 1919[18] ($n = 17$); *T. b. chinensis* Anderson, 1879[5] ($n = 58$); *T. b. clarissa* Thomas, 1917[19] ($n = 43$); *T. b. cochinchinensis* Robinson and Kloss, 1922[15] ($n = 56$); *T. b. concolor* Bonhote, 1907[20] ($n = 33$); *T. b. dissimilis* (Ellis, 1860)[21] ($n = 18$); *T. b. laotum* Thomas, 1914[22] ($n = 37$); *T. b. lepcha* Thomas, 1922[23] ($n = 13$); *T. b. modesta* Allen, 1906[24] ($n = 7$); *T. b. olivacea* Kloss, 1919[18] ($n = 65$); *T. b. siccata* Thomas, 1914[22] ($n = 25$); *T. b. sinus* Kloss, 1916[25] ($n = 2$); *T. b. tenaster* Thomas, 1917[19] ($n = 24$); *T. b. tonquinia* Thomas, 1925[26] ($n = 49$); *T. b. versurae* Thomas, 1922[23] ($n = 15$); *T. b. yunalis* Thomas, 1914[22] ($n = 7$); and *T. g. cognata* Chasen, 1940[27] ($n = 15$). Our data set also included one specimen labeled as *T. b. yaoshanensis* Wang, 1987[28], but this individual was omitted from our analysis due to missing data. When we relabeled by recognized subspecies only, our sample included: *T. b. belangeri* ($n = 511$) and *T. b. chinensis* ($n = 58$).

Taxonomic Results and Discussion

In our PCA of eight skull variables from all available individuals with subspecies labels, PC1 is a size vector with high positive loadings accounting for over 72% of the variation (Supplementary Table S7; Supplementary Fig. S1). PC2, which explains nearly 14% of the variation, represents a

contrast of length variables with negatively weighted width and height variables (Supplementary Table S7; Supplementary Fig. S1). The plot of these two components revealed considerable overlap among subspecies in morphospace (Supplementary Fig. S1). There was no discernible separation among the 21 taxa included in our first plot (Supplementary Fig. S1). Grouping by currently recognized subspecies reveals the *T. b. chinensis* sample to be a subset of the larger and more variable *T. b. belangeri* population (Supplementary Fig. S1).

Based on the extensive infraspecific overlap in skull shape and size, we treat our *T. belangeri* sample as a single population in the rest of our study, and we do not recognize any subspecies. Anderson (1879: 130)[5] originally differentiated *T. b. chinensis* from *T. belangeri* on the basis of its "considerably smaller" skull, and Helgen (2005: 105)[3] chose to recognize *T. b. chinensis* as part of a "simplistic arrangement" to revise the complicated taxonomy of *T. belangeri*. However, here we demonstrate that there is no cranial differentiation between *T. b. chinensis* and other *T. belangeri* populations (Supplementary Fig. S1). Therefore, we do not recognize *T. b. chinensis* as a subspecies, as it is not morphologically distinguishable[29,30] (see Juman et al. 2021[2] for a review of the subspecies concept in treeshrews). Beyond our study, this finding is noteworthy because *T. b. chinensis*, often referred to as the Chinese Treeshrew, is used in biomedical research as an animal model (e.g., Xu et al. 2012[31]). Future research involving *T. belangeri* should take note of this taxonomic revision.

References

1. Sargis, E. J., Woodman, N., Morningstar, N. C., Bell, T. N., & Olson, L. E. Skeletal variation and taxonomic boundaries among mainland and island populations of the common treeshrew (Mammalia: Scandentia: Tupaiidae). *Biol. J. Linn. Soc.* 120, 286–312 (2017).
2. Juman, M. M., Woodman, N., Olson, L.E., & Sargis, E.J. Ecogeographic variation and taxonomic boundaries in Large Treeshrews (Scandentia, Tupaiidae: *Tupaia tana* Raffles, 1821) from Southeast Asia. *J. Mammal.* 102, 1054–1066 (2021).

3. Helgen, K. M. Order Scandentia. In D. E. Wilson, & D. M. Reeder (Eds.), *Mammal species of the world: A taxonomic and geographic reference*, 3rd ed. (Johns Hopkins Univ. Press, 2005).
4. Wagner, J. A. Schreber's Saugthiere, Supplementband, 2. *Abtheilung* 1841 37–44, 553 (1841).
5. Anderson, J. Anatomical and zoological researches: comprising an account of the zoological results of the two expeditions to western Yunnan in 1868 and 1875; and a monograph of the two cetacean genera, *Platanista* and *Orcella* (Quaritch, 1879).
6. Corbet, G. B. & Hill, J. E. The mammals of the Indomalayan region: a systematic review (Oxford University Press, 1992).
7. Sargis E. J., Woodman, N., Reese, A. T., Olson, L. E. Using hand proportions to test taxonomic boundaries within the *Tupaia glis* species complex (Scandentia, Tupaiidae). *J. Mammal.* 94, 183–201 (2013).
8. Sargis, E. J., Woodman, N., Morningstar, N. C., Reese, A. T., & Olson, L. E. Morphological distinctiveness of Javan *Tupaia hypochrysa* (Scandentia, Tupaiidae). *J. Mammal.* 94, 938–947 (2013).
9. Sargis, E. J., Woodman, N., Morningstar, N. C., Reese, A. T., & Olson, L. E. Island history affects faunal composition: The treeshrews (Mammalia: Scandentia: Tupaiidae) from the Mentawai and Batu Islands, Indonesia. *Biol. J. Linn. Soc.* 111, 290–304 (2014).
10. Sargis, E. J., Campbell, K. K., & Olson, L. E. Taxonomic boundaries and craniometric variation in the treeshrews (Scandentia, Tupaiidae) from the Palawan faunal region. *J. Mamm. Evol.* 21, 111–123 (2014).
11. Juman, M. M., Olson, L. E., & Sargis, E. J. Skeletal variation and taxonomic boundaries in the Pen-tailed Treeshrew (Scandentia, Ptilocercidae: *Ptilocercus lowii* Gray, 1848). *J. Mamm. Evol.* 28, 1193–1203 (2021).
12. Juman, M. M., Woodman, N., Miller-Murthy, A., Olson, L.E., & Sargis, E.J. Taxonomic boundaries in Lesser Treeshrews (Scandentia, Tupaiidae: *Tupaia minor* Günther, 1876) *J. Mammal.* doi.org/10.1093/jmammal/gyac080 (2022).
13. R Core Team. *R: A language and environment for statistical computing* (R Foundation for Statistical Computing, 2018).
14. Wickham, H. *ggplot2: elegant graphics for data analysis*. (Springer-Verlag, 2016).
15. Robinson, H. C., Kloss, C. B. New mammals from French Indo-China and Siam. *Ann. Mag. Nat. Hist.* 9, 87–99 (1922).
16. Wroughton, R. C. (A) A new Tree-shrew, (B) A new palm civet from Assam, (C) Assam representative of *O. castaneoventris* groups squirrels. *J. Bombay Nat. Hist. Soc.* 27, 599–601 (1921).
17. Thomas, O. On some small mammals, chiefly bats, from the East Indian Archipelago. *Ann. Mag. Nat. Hist.* 9, 250–255 (1923).
18. Kloss, C. B. On mammals collected in Siam. *J. Nat. Hist. Soc. Siam* 4, 333–407 (1919).
19. Thomas, O. Scientific results from the mammal survey no. XVI. (A) The tupaias of south Tenasserim. *J. Bombay Nat. Hist. Soc.* 25, 199–201 (1917).
20. Bonhote, J. L. On a collection of mammals made by Dr. Vassal in Annam. *Proc. Zool. Soc.* 77, 3–11 (1907).
21. Gray, J. E (Ellis). Early notice of the *Tapaia* [sic] found in Pulo Condore. *Ann. Mag. Nat. Hist.* 3, 71 (1860).

22. Thomas, O. The Tree-Shrews of the *Tupaia belangeri-chinensis* group. *Ann. Mag. Nat. Hist.* 8, 243–245 (1914).
23. Thomas, O. On mammals from the Yunnan Highlands collected by Mr. George Forrest and presented to the British Museum by Col. Stephenson R. Clarke, D.S.O. *Ann. Mag. Nat. Hist.* 9, 391–406 (1922).
24. Allen, J. A. Mammals from the Island of Hainan, China. *Bull. Am. Mus. Nat. Hist.* 22, 463–491 (1906).
25. Kloss, C. B. On a collection of mammals from the coast and islands of South-East Siam. *Proc. Zool. Soc.* 86, 27–76 (1916).
26. Thomas, O. The Mammals obtained by Mr. Herbert Stevens on the Sladen-Godman Expedition to Tonkin. *Proc. Zool. Soc.* 95, 495–506 (1925).
27. Chasen, F. N. A handlist of Malaysian mammals (A systematic list of the mammals of the Malay Peninsula, Sumatra, Borneo and Java, including the adjacent small islands). *Raffles Bull. Zool.* 15, 1–209 (1940).
28. Wang, Y. X. Taxonomic research on Burma-Chinese tree shrew, *Tupaia belangeri* (Wagner), from southern China. *Zool. Res.* 8, 213–230 (1987).
29. Mayr, E. & Ashlock, P. D. Principles of systematic zoology (McGraw-Hill, 1991).
30. Braby, M., Eastwood, R., & Murray, N. The subspecies concept in butterflies: has its application in taxonomy and conservation biology outlived its usefulness? *Biol. J. Linn. Soc.* 106, 699–716 (2012).
31. Xu, L., Chen, S. Y., Nie, W. H., Jiang, X. L., Yao, Y. G. Evaluating the phylogenetic position of Chinese tree shrew (*Tupaia belangeri chinensis*) based on complete mitochondrial genome: implication for using tree shrew as an alternative experimental animal to primates in biomedical research. *J. Genet. Genomics* 39, 131–137 (2012).
32. Bivand, R. S., Pebesma, E. & Gomez-Rubio, V. *Applied spatial data analysis with R, Second edition* (Springer, 2013).

Appendix

Specimens examined

Mainland (*n* = 726)

BANGLADESH (*n* = 5). Tangail: Thana Madhupur: Jalchatra (SMF 59045, 59046, 59047, 59048, 59049).

BHUTAN (*n* = 6). Duars: Hasimara (NHMUK 16.7.29.43, 16.7.29.44, 16.7.29.45, 16.7.29.46); Bharnabhari (NHMUK 16.7.29.47, 16.7.29.48).

CAMBODIA (*n* = 16). no locality (MNHN 1982-50, 1982-51, 1980-264); Bokor (NHMUK 28.7.1.26, 28.7.1.27); Kampot (MNHN 1971-804; USNM 321548); Kompong-Chnang [Kampong Chhnang] (MNHN 1982-48); Ok Yam (NHMUK 15.11.4.35, 15.11.4.36); Sambor (NHMUK 28.7.1.23); Siem Reap (NHMUK 28.7.1.22; MNHN 1929-370, 1929-371); Tuk Sap (MNHN 1982-49, 1982-769).

CHINA (*n* = 42). Tengyueh, Ta Chupa (NHMUK 13.4.29.1); Tibet, Yer-Ka-Lo [Yerkalo] (MNHN 1877-173); Yaoshan, Kuangsi [Guangxi] (ZMB 45384). Yunnan Province (NHMUK 1996.490, 1996.491, 1997.388, 12.7.25.15, 12.7.25.16, 12.7.25.17, 12.7.25.18, 12.7.25.19, 12.7.25.45, 24.5.10.1; USNM 240120): Dali, Erhai Lake (MCZ 20686; AMNH M-44292); east of Lichiang Valley (NHMUK 22.9.1.9); Tengyueh (NHMUK 25.10.5.2; 25.10.5.3; 26.5.11.5); Kao Chiao (MCZ 24497; AMNH M-84940); Li Kiang Plain (USNM 240117); Lichiang Range (22.9.1.4, 22.9.1.5, 22.9.1.8, 23.4.1.5, 23.4.1.7); Lijiang, Dayan (FMNH 33956); Mee Chee (NHMUK 8.11.14.14); Mongtze (MCZ 13686); Milati (NHMUK 21.9.1.2); Mucheng, Salween Drainage (AMNH M-44311); Nanting [Namting] River (AMNH M-44315); Shuichai, Mekong River (AMNH M-44291); Shweli [Ruili] Valley (NHMUK 26.5.11.3); Tsao Chiang (USNM 240119); Wai-ta, Mekong River (MCZ 20684); Yun Lung (USNM 240118); Yung-chang-fu (NHMUK 14.10.23.1); Yunnan Fu (AMNH M-84934); Yunnanyi (FMNH 39290).

INDIA (*n* = 50). Arunachal Pradesh: Mishmi Hills: Dreyi (NHMUK 21.12.5.13). Assam: Ledo (USNM 279321); Cachar, Dilkhush (NHMUK 82.2.6.3); Lohit Valley, Sadiya (NHMUK 20.6.7.10, 20.6.7.11); Imphal (NHMUK 67.60); Karong (FMNH 76140); Mishmi Hills (AMNH M-163088); Mishmi Hills, Dening (NHMUK 21.12.5.9, 21.12.5.10, 21.12.5.11, 21.12.5.12; FMNH 82594, 82595); Naga Hills (NHMUK 47.306, 47.307, 66.2867). Bengal: Sangsir (USNM 260739). Darjeeling: Ghoom (NHMUK 15.9.1.43); Nurbong (NHMUK 15.9.1.44, 20.6.22.2); Reang (NHMUK 26.10.8.16). Manipur: Imphal (USNM 279178); Aimole (NHMUK 85.8.18.8); Haingyaw (NHMUK 15.5.5.36); Machi (NHMUK 85.8.18.9); Tsibit (NHMUK 15.5.5.35). Meghalaya: Dura Banda (NHMUK 20.11.1.26); Dura Giri (NHMUK 20.11.1.27; FMNH 82592); Mt. Tura (FMNH 76146); Tura (NHMUK 20.11.1.24, 20.11.1.25; AMNH M-26843); Cherrapunji (FMNH 76135, 76137); Laitkynsao (FMNH 82593); Mawryngkueng (FMNH 76144). Mizoram: Lushai Hills: Sangao (FMNH 76151, 76152, 76153, 76156, 76157, 76158). Nagaland: Naga Hills: Kohima (FMNH 76145); Mokokchung (NHMUK 20.6.6.3). Sikkim (NHMUK 86.11.15.1): Barnyak (MCZ 1923); Mangpu (FMNH 34784, 35427).

LAOS (*n* = 48). Khammouane (ROM 106402); Lo Tiao (MCZ 38269, 38270, 38271, 38272); Myong Yo (FMNH 32413); Muong, Soui (MCZ 38273); Nam Khueng (MCZ 38267); Nape (MNHN 1929-358, 1929-360; NHMUK 28.7.1.28); Pakse (FMNH 37881, 37882, 37971, 37972, 37975); Phong Saly (FMNH 32407, 32410, 32411, 32412); Plateau Bolovens (AMNH M-87322, M-87323, M-87327, M-87330, M-87333, M-87334, M-87337, M-87338, M-87339, M-87341, M-87342, M-87345); Saravane, Thateng (USNM 260738). Xien Luang-Koo [Xiangkhouang Province] (MNHN 1929-354, 1929-361, 1929-362, 1929-363, 1929-364, 1929-365, 1929-366, 1929-369, 1947-643; NHMUK 26.10.4.23, 26.10.4.24, 26.10.4.26, 26.10.4.27): Ban Theuong (USNM 355452, 355453).

MYANMAR (*n* = 115). Amherst, Tichera (NHMUK 6.7.5.2); Assoon (NHMUK 85.8.1.83); Bago, Dawe (USNM 583794, 583795, 583796); Banlaw, Tenasserim River (NHMUK 14.12.8.93); Chindwin (NHMUK 78.2531, 15.5.5.37); Dalu (AMNH M-113132, M-113133); Gangfang (AMNH M-114867, M-114868, M-114870, M-114871); Haibum (M-113135); Hat Sanuk (NHMUK 55.1304, 55.1305, 55.1306, 55.1307); Hekamti (NHMUK 32.11.1.8); Htawgaw (AMNH M-114875); Htingnan (NHMUK 50.488); Kuakargit [Kyauksarit] (ZMB 5508); Kyauk Myaung, Irrawaddy (NHMUK 14.7.19.84); Lonkin (AMNH M-113122, M-113123, M-113127); Ma-chang Kai, Tengyueh (NHMUK 12.8.26.1, 13.12.8.4); Madaya (NHMUK 36.12.26.8); Mandalay, Pyin-Oo-Lawin, National Kandawgyi Gardens (USNM 584375, 584376); Mansum-Gora [Ghura] (AMNH M-113124, M-113125, M-113126, M-113129); Mawlamyine, Moulmein (NHMUK 85.8.1.80); Maymyo (ZMB 90818, 90819, 90820); Meiktila, Pyawbwe (NHMUK 14.7.19.81); Mingun, West Sagaing (NHMUK 14.7.19.73, 14.7.19.74); Mon, Telok Besar (FMNH 43838); Mt. Popa (ZMB 90778, 90780, 90781; NHMUK 14.7.19.75, 14.7.19.77, 14.7.19.78, 14.7.19.79); Myitkyina (LACM 8157; KU 16208); Nam Tamai Valley (NHMUK 1938.5.5.1); Nanyaseik (AMNH M-113118, M-113119, M-113120, M-113121); Pagan (NHMUK 14.7.19.85). Prome [Pyay] (ZMB 90769); Pumsin (AMNH M-113130, M-113131); Pyaunggaung (NHMUK 14.7.8.8); Pyintha Taung [Puim-La-Taung] (AMNH M-143830); Rangoon (NHMUK 6.4.5.3, 7.7.20.7); Sagain (USNM 577421, 577422, 577423, 577424); Se-eng [Seien] (NHMUK 14.7.8.9); Sima, Kyitkyina (NHMUK 9.7.20.1); Tagool, Tenasserim River (NHMUK 17.3.25.3, 17.3.25.4); Tamu (AMNH M-114865); Tatkon (NHMUK 15.5.5.33, 15.5.5.34); Thaungyin (NHMUK 33.7.9.1); Zibugaung (NHMUK 6.7.5.1). Pegu (NHMUK 17.4.24.8, 17.4.24.9, 63.5.9.11): Bamalik Chaung (AMNH M-54779); Camp Pinmezali (AMNH M-54775); Yetho River (AMNH M-54788, M-54797). Tenasserim (SMF 4944; NHMUK 85.8.1.78, 85.8.1.79): Bankachon (NHMUK 14.12.8.96, 14.12.8.97, 14.12.8.98, 14.12.8.99, 14.12.8.100, 17.3.25.1); Bokpyin (USNM 104358); Lakya (NHMUK 24.9.2.20); Meetan (NHMUK 85.8.1.81); North Tenasserim (NHMUK 88.6.18.1); South Tenasserim, Victoria Point (NHMUK 78.2525; USNM 124003); Tanjong Badak (USNM 104357); Tavoy (NHMUK 85.8.1.84); Telok Besar (USNM 124284); Tenasserim Town (NHMUK 14.12.8.94, 14.12.8.95, 17.3.25.2); Thaton (NHMUK 85.8.1.85); Toak Plateau (AMNH M-54699); Toungoo (NHMUK 27.11.18.11, 27.11.18.12, 36.12.26.9; USNM 20888; FMNH 82585, 82587).

NEPAL (*n* = 2). Chatra (USNM 290063); Haraincha, Morang (NHMUK 1938.8.2.1).

PAKISTAN (*n* = 1). Chittagong Hills: Manimukh: Mahallya (MCZ 57921).

THAILAND (n = 269). Bangkok (FMNH 34116, 47344; KU 125481; MCZ 23316, 23318, 23319, 23321, 23324, 23325, 23326, 23327, 57548, 57549, 57550, 57552, 57554, 57555, 57556; MVZ 119721; NHMUK 55.1312, 55.1313, 55.1314; UMMZ 58977, 58978, 58979, 58980, 58981, 58982; USNM 240046, 241058, 241059, 241061, 241062, 241063, 257819; ZMB 41073, 41074, 41075, 41076, 41077, 41079, 41082, 41083, 41084, 41085, 41670; ZMUC 734, 735); Chaiyaphum, Phu Khiao (USNM 294844, 294846, 294848, 294845); Chanthaburi (USNM 258926, 258929, 535150, 535151); Chiang Rai, Chian Saen Kao (USNM 296885); Chonburi (USNM 241065, 241453, 256885, 296912); Chumporn (AMNH M-250028, M-250029); Doi Inthanon, Ang Ka Peak (MCZ 35810, 35812, 35813, 35814, 35815, 35816, 35817, 35818, 35819, 35820, 35821, 35823, 35824, 35825, 35827, 35829, 35830, 35837, 35838, 35839, 35840, 35841, 35842; YPM MAM 310, 311); Hoa Hin (NHMUK 15.12.20.1); Kamphaeng Phet (FMNH 66121, 66123, 66125; USNM 296886, 296887, 296888); Kanchanaburi (USNM 296869, 296870, 296872, 296873, 296875, 296876, 253448); Khao Soi Dao (MNHN 1990-501 1990-502, 1990-503, 1990-504, 1990-505, 1990-506, 1990-507, 1990-508, 1990-509); Kohn Kaen, Chum Phae (USNM 355020, 355021, 355022, 355023, 355024, 355025, 355026, 355027, 355028, 355029, 355030, 355031, 355032, 355033, 355034, 355037, 355038); Klong Mennau (NHMUK 15.11.4.37); Klong Yai (NHMUK 15.11.4.32, 15.11.4.33, 15.11.4.34); Koharut [Khao Krut] (NHMUK 1939.3764); Lampang, Pang La (USNM 296884); Lamphun, Khun Tan (USNM 257817); Loei, Dan Sai (USNM 300013, 307691, 307692, 307699, 307700, 307701, 307702, 307704, 307705, 307706, 307707, 307708, 307709); Lopburi (USNM 296908, 296911); Mae Hong Son (USNM 267229); Maprit, Patiyu (NHMUK 20.7.3.10, 20.7.3.11); Mewong River (AMNH M-54714, M-54719, M-54821; NHMUK 24.9.2.18, 24.9.2.19); Muang Na (MNHN 1990-511); Muang Pai (NHMUK 9.10.11.10, 9.10.11.11); Muongsen (NHMUK 26.10.4.29, 26.10.4.30); Nongkok, Grabi (NHMUK 55.1278, 55.1279, 55.1280, 55.1281, 55.1282, 55.1283, 55.1284, 55.1285, 55.1286, 55.1287); Prachuap Khiri Khan, Ban Khlua Klang (USNM 296877, 296878, 296879, 296880, 296881, 296882, 296883); Rahen, Tak (USNM 253566); Raheng (NHMUK 7.11.13.3); Renong River (NHMUK 55.1295); Southern Line (NHMUK 19.8.7.1, 19.8.7.2); Surat Thani, Tha Chang (CMNH 87906, 87907); Tang Prau, Tahuatung (NHMUK 55.1299); Tapli, Pakchan (NHMUK 55.1296, 55.1297, 55.1298); Chumpawn (NHMUK 55.1288, 55.1289, 55.1290, 55.1291, 55.1292, 55.1293, 55.1294); Ubon Ratchathani, Phibun Mangsahan (USNM 355050, 355053). Chiang Mai (NHMUK 98.10.5.3, 98.10.5.4, 98.10.5.5, 98.10.5.6; USNM 260631): Chieng Dao (MCZ 35828; USNM 257816); Hod (USNM 355018); Pha Lad (USNM 355007, 355008, 355010, 355011, 355013). Ko Lak (NHMUK 19.2.9.1, 19.2.9.2, 19.2.9.3): Prachuap Khiri Khan (USNM 221576, 221577, 221578); Rajburi (NHMUK 55.1309, 55.1310, 55.1311). Nakhon Ratchasima: Amphoe Pak Chong (USNM 251693); Amphoe Pak Thong Chai (USNM 241452); Korat (ROM 34233); Lat Bua Kao (USNM 221574, 254757); Pak Chong (USNM 355041); Sikhiu (USNM 296890). Nakhon Sawan: Kow Chong Sala Mountain (USNM 296897, 296898); Pak Nam Pho (USNM 296891, 296892, 296893, 296894, 296895, 296899, 296900, 296902, 296905, 296906, 296907). Nan (NHMUK 98.2.8.12): Ban Nam Kien (USNM 255758); Ban Pha Hang (USNM 355046, 355047, 355048); Doi Phu Kha (USNM 261086, 261087). Nongbua Lamphu: Ban Chieng Pin (USNM 355057, 355058); Ban Gud Ling Khor (USNM 355059, 355060). Ratchaburi: Jombung, Tung Narkarien (USNM 294849, 294850, 296512); Pak Tho (USNM 296510). Sakon Nakhon: Ban Khok Phu, Ban Sang Kho (USNM 300012); Phu Phan (USNM 307684); Tachin (USNM 221561, 221562). Trat: Amphoe Khao Saming (USNM 535152, 256884); Bang Kradan (USNM 396252, 396253, 396256); Klong Yai (FMNH 47345; USNM 201431); Ok Yam (USNM 201430).

VIETNAM (*n* = 172). Bac Tan Trai (FMNH 32402); Bac-Kan (NHMUK 27.12.1.54, 27.12.1.55, 27.12.1.57, 27.12.1.59); Bana (MNHN 1960-3857); Bao-Ha (NHMUK 25.1.1.22); Bien Hoa (USNM 258008); Binh Thuan, Song Trao (USNM 320712, 320713, 320714); Cap St. Jacques [Vung Tau] (FMNH 38871); Chapa (FMNH 38900, 38902, 38904; MCZ 26943, 38263, 38264; NHMUK 33.4.1.137, 33.4.1.138); Cho Ba (NHMUK 27.12.1.61); Danang (ROM 86975); Ho Chi Minh City (MNHN 1961-756); Lai Chau (USNM 240500, 240501); Lien San (FMNH 32404, 32405); Long Khanh, Caryu Danar (USNM 320770, 320771, 320772); Lung Lunh (FMNH 38908); Muong Boum (FMNH 32403); Nam He (FMNH 32406); Ngai-Tio (NHMUK 25.1.1.19); Pa Ham (FMNH 32399); Phluoc Tuy, Phuoc Hai (USNM 320773, 320774); Phong Tho (FMNH 32401); Quang Tri, Vinh Linh (MNHN 1929-368); Quang Tin, Chu Lai (USNM 356398, 356399, 356401,356971, 356973, 356975); Quang Tri, Calu (USNM 357642, 357643, 357644); Sontay, Mt. Bavi (NHMUK 54.2, 54.3); South Annam, Da Ban (NHMUK 26.11.17.2); Tayninh (NHMUK 28.7.1.24, 28.7.1.25); Thai-Nien (NHMUK 25.1.1.18); Tonkin (MCZ 13685; MNHN 1898-1596); Trangbom (NHMUK 26.11.17.1); Yen Bai (LACM IH 33, IH 85). Annam: Dak-To (NHMUK 26.10.4.32, 26.10.4.33, 26.10.4.34); Eaktur (FMNH 46642); Hoi-Xuan (FMNH 38905, 38907; NHMUK 33.4.1.141, 33.4.1.142, 33.4.1.143, 33.4.1.144, 33.4.1.145); Kontoum (NHMUK 26.10.4.35); Nhatrang (NHMUK 6.11.6.3); Phu Zui (NHMUK 28.7.1.29, 28.7.1.30); Thua-Luu (NHMUK 27.12.1.53, 27.12.1.60); Darlak: M'drak (USNM 320762); Ban Me Thout (USNM 320765, 320766, 320767, 320768, 320769). Khanh Hoa: Ninh Ma (USNM 320744, 320745, 320746, 320751, 320757); Xuan Phu (USNM 320741, 320743); Dai Lanh (USNM 320748); Phu Xuong (USNM 320739, 320740). Lam Dong: Blao (USNM 320683, 320690, 320691, 320695, 320696, 320697, 320698, 320702, 320704, 320706, 320686, 320687, 320701); Dijiring (USNM 320641, 320660, 320663, 320664, 320667, 320669, 320670, 320673, 320676, 320681, 320682, 355448). Ninh Thuan: Krong Pha (USNM 320727, 320732); Tan My (USNM 320715, 320716, 320722, 320723, 320724, 320729, 320730, 320733, 320737); Ap Hung Lam (USNM 320655, 320656, 320657, 320662, 320643, 320649, 320650); Nha Ho (USNM 320734, 320735, 320736). Quang Nam: Da Nang (USNM 357995, 357996, 357997, 357999, 358000, 358002, 358004, 358005, 358006); Mt. Sontra (USNM 356381, 356383, 356384, 356385, 356386, 356387, 356389, 356390, 356394, 356397, 356970, 357580, 357765, 357879, 357993, 356388, 357582, 356391, 356392, 356393, 357581). Tuyen Duc: Dalat (USNM 320638, 320640); Fyan (USNM 320775, 320777).

Islands (*n* = 111)

CHINA (*n* = 20). Hainan: Cheonkhon [Cheongkong] (NHMUK 85.8.1.87); Hoi How (AMNH M-26658); Kachek (USNM 239286); Lei-Mui-Mon (AMNH M-26654, M-26655, M-26656, M-26657, M-26660); Nam Fong (AMNH M-59840); Nodoa (AMNH M-55988, M-59828, M-59837, M-59838, M-59839, M-59844, M-59846, M-59848; MCZ 24498, 24499); Utoshi (AMNH M-26659).

MYANMAR (*n* = 44). Mergui Archipelago: Bentinck Island (USNM 104364); Clara Island (USNM 124143); Domel Island [Letsok-aw Kyun] (USNM 104363, 124173); Hastings Island (NHMUK 23.1.6.29, 23.1.6.30); James Island [Pulo Buda] (USNM 124076, 124077, 124078, 124079, 124080); King Island [Kadan Kyun] (NHMUK 23.1.6.35, 23.1.6.36, 23.1.6.37, 82598); Kisseraing Island [Kanmaw Kyun] (NHMUK 23.1.6.33, 23.1.6.34; USNM 124202, 124203,

124204); Malcolm Island (FMNH 82602; NHMUK 23.1.6.31); Ross Island [Daung Kyun] (NHMUK 23.1.6.44, 23.1.6.45, 23.1.6.46, 23.1.6.47, 23.1.6.48, 23.1.6.49; FMNH 82601); Sir John Hayes Island [Kunthi Kyun] (NHMUK 23.1.6.32); St. Luke's Island [Zadetkalay] (USNM 104362); St. Matthew's Island [Zadetkyi Kyun] (USNM 104359, 104360, 104361, 104400, 111911); Sullivan Island [Lanbi Kyun] (USNM 124102, 124103); Tavoy Island [Mali Kyun] (NHMUK 23.1.6.38, 23.1.6.39, 23.1.6.40, 23.1.6.41, 23.1.6.42, 23.1.6.43).

THAILAND (*n* = 24). Ko Lanta (NHMUK 55.1367, 55.1369, 55.1371, 55.1373); Ko Ra (NHMUK 55.1303); Koh Chang (NHMUK 15.11.4.29, 15.11.4.30, 15.11.4.31, 201435, 201436); Panjang Island [Ko Yao Yai] (NHMUK 47.1495, 55.1356, 55.1357, 55.1358, 55.1359, 55.1361, 55.1362, 55.1363, 55.1364, 55.1365, 55.1366); Phuket (NHMUK 55.1300, 55.1301, 55.1302).

VIETNAM (*n* = 23). Con Son Island (USNM 357253, 357252, 357251, 357254, 357004, 357006, 357005, 357003, 357350; MNHN 1882-10; NHMUK 55.1404, 55.1405, 55.1406, 55.1407; ZMB 3745); Ben Dam (USNM 357189, 357188, 357191); Nui Chua (USNM 357247, 357249); Nui Nha Ban (USNM 357192, 357193, 357250).

No Locality (*n* = 2)

CHINA (ZMB 87167); MYANMAR (NHMUK 62.7.16.12).

Supplementary Tables

Supplementary Table S1. First principal component (PC1) loadings for the 18 skull measurements. Abbreviations for measurements are defined in Supplementary Table S5.

**Measurement**	**PC1**
CPL	0.9743
LPL	0.9732
MCIL	0.9692
CNL	0.9540
PPL	0.9420
UTL	0.9318
PBPL	0.9206
LTL	0.9133
LTPL	0.8917
MTL	0.8782
ZB	0.8317
LIB	0.8244
LCH	0.8075
BB	0.7752
MH	0.7640
MCH	0.7389
MB	0.7336
MCW	0.7247
Eigenvalue	13.567
% variance	75.370

Supplementary Table S2. Effect of Sex, Latitude, Source of the population (island or mainland), and Collection Year on PC1; estimate, standard error (*SE*), t statistics (*t* value), and significance level (*p*r[>*t*]).

	Estimate	*SE*	*t* value	*p*r(>*t*)
Sex (Male)	2.11	0.23	9.18	< 0.0001
Latitude	-2.07	0.13	-15.96	< 0.0001
Source (Island)	0.56	1.02	0.55	0.5800
Collection Year	0.59	0.12	4.93	< 0.0001
Latitude x Source (Island)	2.56	0.59	4.36	< 0.0001
Sex (Male) x Source (Island)	-1.42	0.64	-2.23	0.0259
Collection Year x Latitude	-0.89	0.14	-6.49	< 0.0001
Collection Year x Source (Island)	-1.38	0.88	-1.55	0.1206
Collection Year x Latitude x Source (Island)	1.29	0.54	2.39	0.0172

Supplementary Table S3. Hierarchical partitioning analysis with the independent contribution to variance of each variable (I obs), the percent variance explained (I %), Z score obtained from 10,000 iterations, and associated significance; *: $p < 0.05$, ns: $p > 0.05$.

	I obs	I %	Z score
Mainland Distance	0.18	57.02	13.56*
Latitude	0.05	14.72	2.74*
Sea Depth	0.04	13.25	2.42*
Island Area	0.03	9.81	1.64^{ns}
Sex	0.02	5.20	0.52^{ns}

Supplementary Table S4. Effect of Sex, Temperature, Precipitation, Primary Forest Cover, and Urban Proximity on PC1; estimate, standard error (*SE*), *z* value, and significance level (*p*r[>*z*]).

	Estimate	*SE*	*z* value	*p*r(>*z*)
Sex (Male)	2.09	0.18	11.84	< 0.0001
Temperature	0.52	0.17	3.02	0.0025
Precipitation	0.10	0.10	1.08	0.2806
Elevation	0.18	0.12	1.53	0.1269
Primary Forest Cover	-0.00	0.17	-0.03	0.9792
Total Forest Cover	0.18	0.18	1.02	0.3069
Urban Proximity	0.03	0.10	0.25	0.8016
Temperature x Precipitation	0.18	0.09	2.12	0.0337

Supplementary Table S5. Cranial and mandibular measurement descriptions (and abbreviations) following Sargis et al. (2013, 2014a, 2014b, 2017, 2018), and Juman et al. (2021a, 2021b, 2022). Uppercase abbreviations (i.e., I, C, P, M) refer to maxillary and premaxillary teeth; lowercase abbreviations (i, c, p, m) refer to mandibular teeth.

Cranium

Braincase breadth (BB): greatest breadth of braincase.
Condylo-incisive length (CIL): greatest distance between anterior-most surface of I1 and caudal surface of occipital condyle.
Condylo-nasal length (CNL): greatest distance between rostral surface of nasal and caudal surface of occipital condyle.
Condylo-premaxillary length (CPL): greatest distance between rostral surface of premaxilla and caudal surface of occipital condyle.
Epipterygoid breadth (EB): greatest distance between lateral points of epipterygoid processes.
Epipterygoid-premaxillary length (EPL): greatest distance between rostral surface of premaxilla and caudal surface of epipterygoid process.
Lacrimal breadth (LB): greatest distance between lateral apices of lacrimal tubercles.
Lambdoid crest height (LCH): greatest distance from apex (or apices if bilobate) of lambdoid crest to both ventral apices of occipital condyles (i.e., along midline).
Least interorbital breadth (LIB): least distance between the orbits.
Lambdoid-premaxillary length (LPL): greatest distance between rostral surface of premaxilla and caudal surface of lambdoid crest.
Lacrimal tubercle-premaxillary length (LTPL): greatest distance between rostral surface of premaxilla and caudal surface of lacrimal tubercle.
Mastoid breadth (MB): greatest distance between lateral apices of mastoid portion of petrosal.
Maxillary toothrow length (MTL): greatest distance between anterior-most surface of C1 and posterior-most surface of M3.
Postorbital bar-premaxillary length (PBPL): greatest distance between rostral surface of premaxilla and caudal surface of postorbital bar.
Palato-premaxillary length (PPL): greatest distance between rostral surface of premaxilla and caudal surface of palatine.
Upper toothrow length (UTL): greatest distance between anterior-most surface of I1 and posterior-most surface of M3.
Zygomatic breadth (ZB): greatest distance between lateral surfaces of zygomatic arch.

Mandible

Lower toothrow length (LTL): greatest distance between anterior-most surface of i1 and posterior-most surface of m3.
Mandibular condyle height (MCH): greatest distance between mandibular condyle and angular process of mandible.
Mandibular condylo-incisive length (MCIL): greatest distance between anterior-most surface of i1 and caudal surface of mandibular condyle.
Mandibular condyle width (MCW): greatest distance between medial and lateral surfaces of mandibular condyle.
Mandibular height (MH): greatest distance between coronoid and angular processes of mandible.

Supplementary Table S6. Latitude (N), island area (km^2), distance to mainland (km), and maximum sea depth (m) for islands.

Island	Latitude	Island area	Distance to mainland	Maximum sea depth[c]
Hainan	19.00–20.05	33209.8[a]	20.0[a]	108
Koh Chang	12.00–12.06	214.6[a]	5.0[a]	31
Mergui Archipelago				
Mali Kyun	13.10	99.3[a]	30.0[a]	107
Kadan Kyun	12.49	449.8[a]	10.0[a]	61
Daung Kyun (Don)	12.21	110.2[a]	45.0[a]	39
Kunthi Kyun	11.8	23.1[b]	101.5[b]	102
Bentinck	11.75	77.9[a]	60.0[a]	79
Kanmaw Kyun	11.67	408.8[a]	5.0[a]	30
Letsok-aw Kyun	11.62	249.9[a]	35.0[a]	50
(Sir J.) Malcolm	11.3	23.2[b]	50.8[b]	54
Clara	10.9	37.4[b]	68.3[b]	68
Lanbi Kyun	10.83	187.8[a]	20.0[a]	24
Pulo Buda	10.5	20.0[b]	27.6[b]	33
Zadetkalay	10.1	44.8[b]	30.8[b]	35
Hastings	10.1	14.5[b]	22.0[b]	28
Zadetkyi Kyun	9.97	176.2[a]	25.0[a]	41
Ko Ra (Rak)	9.97	19.3[b]	3.0[b]	15
Côn Sơn	8.65–8.70	58.9[b]	84.2[b]	43
Phuket	8.05	543.1[b]	1.0[a]	8
Ko Yao Yai	8.00	89.4[b]	15.0[b]	30
Ko Lanta	7.58	81.0[b]	1.0[a]	0

[a]http://islands.unep.ch/isldir.htm
[b]Google Earth version 9.156.0.0
[c]GeoMapApp 3.6.14 (www.geomapapp.org 2021; Ryan et al. 2009)

Supplementary Table S7. Principal component (PC) loadings for the 8 skull variables included in the taxonomic principal component analysis (Supplementary Fig. S1). Abbreviations for measurements are defined in Supplementary Table 5.

**Measurement**	**PC1**	**PC2**
UTL	0.9248	0.3115
PBPL	0.9202	0.3409
LTPL	0.8944	0.3766
MTL	0.8727	0.2899
LIB	0.8376	-0.1663
MH	0.8031	-0.4909
MCH	0.7909	-0.4568
MCW	0.7441	-0.3988
Eigenvalue	5.789	1.075
% variance	72.366	13.443

Supplementary Figures

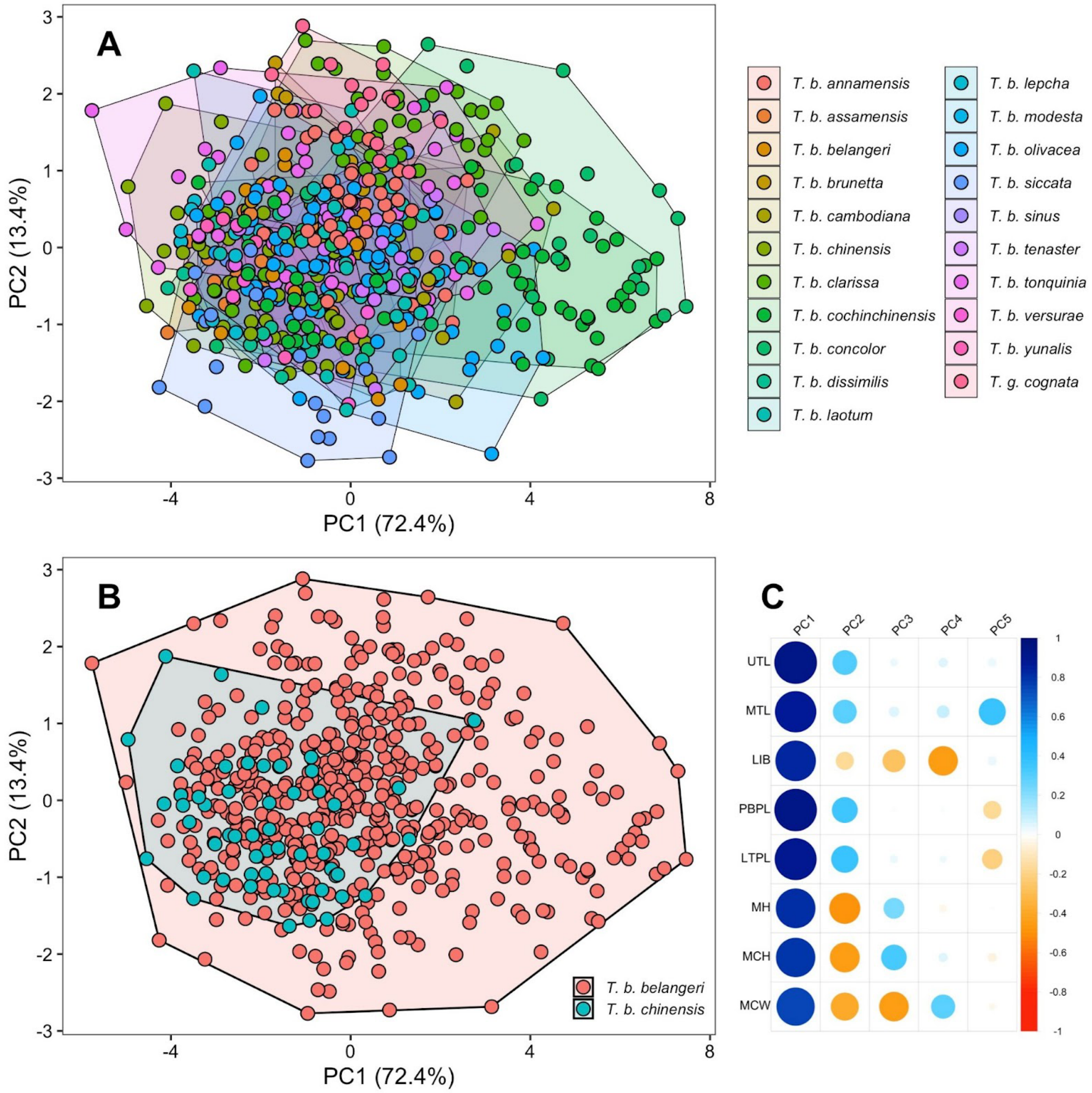

Supplementary Fig. S1: Principal component analysis (PCA) depicting morphometric overlap among *T. belangeri* subspecies. (A) Bivariate plot of PC scores on the first two axes from PCA of eight skull variables, color-coded by the 21 taxa represented in this sample. (B) Plot of PC scores, color-coded by the two currently recognized subspecies: *T. b. belangeri* and *T. b. chinensis*[27]. (C) Loadings for each of the eight skull measurements on the first five PCs (Supplementary Table S7).

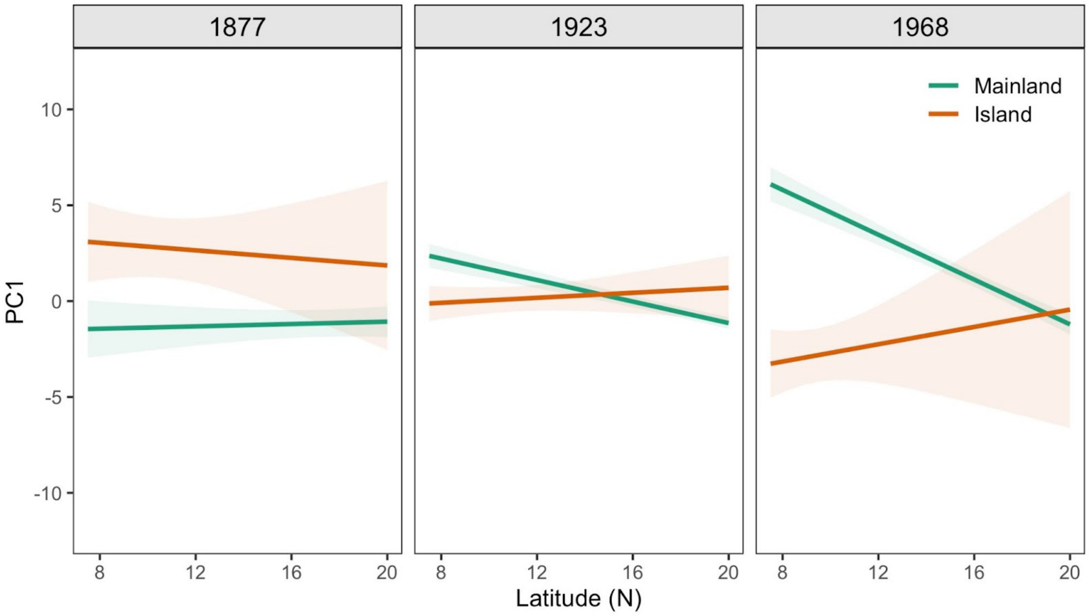

Supplementary Fig. S2. A plot depicting the positive interaction between Latitude, Collection Year, and Source (Island) in a linear model predicting PC1 (Supplementary Table S2). Regression lines are displayed at three evenly spaced intervals spanning the temporal range of the island sample and plotted across the latitudinal range of the island sample with 95% confidence intervals.

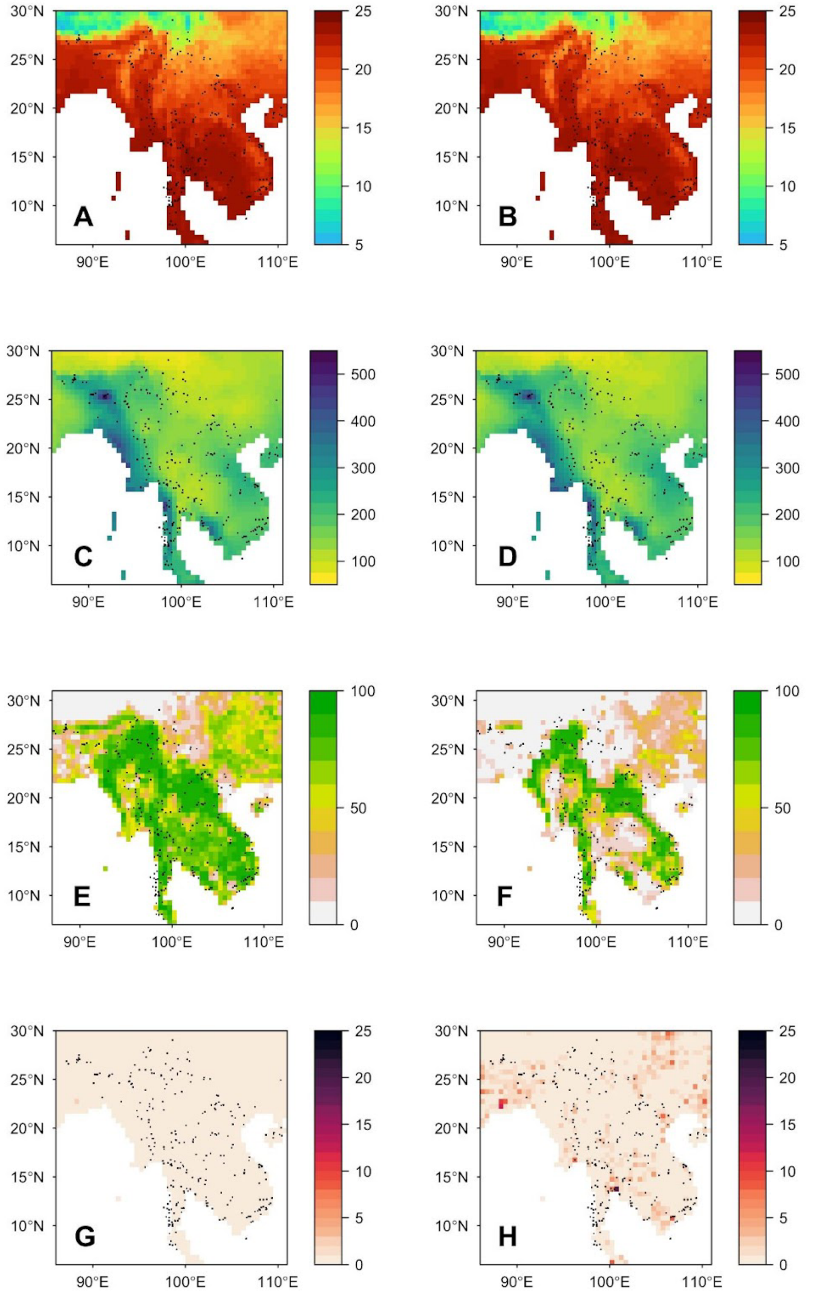

Supplementary Fig. S3: Maps depicting environmental conditions in our study area at the earliest and latest years (within the temporal range of our data set) for which historical data are available at 0.5° x 0.5° gridded resolution. Black dots on each map indicate localities included in our study. Maps were generated using the package *sp*[32] in R (version 3.5.1)[13]. (A) Average annual temperature (°C), 1901-1905. (B) Average annual temperature (°C), 1999-2003. (C) Average

annual precipitation (cm), 1901-1905. (D) Average annual precipitation (cm), 1999-2003. (E) Percentage of primary forest cover per cell, 1874. (F) Percentage of primary forest cover per cell, 2003. (G) Percentage of urban land use per cell, 1874. (H) Percentage of urban land use per cell, 2003.
